# Anesthetic and analgesic management of Svalbard rock ptarmigan (*Lagopus muta hyperborea*) undergoing experimental stereotaxic neurosurgery

**DOI:** 10.1186/s12917-025-05082-3

**Published:** 2025-10-31

**Authors:** Jana Kalinová, Daniel Appenroth, Fernando Cázarez-Márquez, Renate Thorvaldsen, David G. Hazlerigg, Ingebjørg H. Nymo

**Affiliations:** 1 Arctic Seasonal Timekeeping Initiative (ASTI), Department of Arctic and Marine Biology, Arctic Chronobiology and Physiology Research Group, UiT – The Arctic University of Norway, Tromsø, Norway; 2 Department of Arctic and Marine Biology, Arctic Chronobiology and Physiology Research Group, UiT – The Arctic University of Norway, Tromsø, Norway; 3 Department of Animal Health, Welfare and Food Safety, Norwegian Veterinary Institute, Tromsø, Norway

**Keywords:** Animal welfare, Bupivacaine, Buprenorphine, Isoflurane, Meloxicam, Multimodal analgesia, Refine, Stereotaxic neurosurgery

## Abstract

**Background:**

Stereotaxic neurosurgery in animal models has become a prominent experimental technique in many areas of science and research. These approaches, however, require advanced surgical skills, meticulous anesthetic protocols, and thorough analgesic management to ensure scientific accuracy and animal welfare. The specialized anatomy and physiology of birds, combined with high interspecies variability, make the use of anesthetics, analgesics, and other medications particularly challenging, especially in the absence of well-established guidelines. Here, we present an anesthetic-analgesic protocol developed for stereotaxic neurosurgery for neuronal tracer injection in Svalbard rock ptarmigan (*Lagopus muta hyperborea*).

**Results:**

6 Svalbard rock ptarmigan underwent stereotaxic neurosurgery and a multimodal anesthetic-analgesic approach was developed, including isoflurane (inhalation, induced with 3–5%, maintained with 1–3%), bupivacaine (2 mg/kg s.c. during anaesthesia, preoperatively), buprenorphine (0.05 mg/kg i.m. during anaesthesia, both preoperatively and postoperatively), and meloxicam (0.4 mg/kg i.m. during anaesthesia, postoperatively, followed by 0.4 mg/kg p.o. q24h until euthanasia on day 7). Birds were monitored during surgery for vital signs. Post-mortem analysis was performed to confirm neuronal tracer placement. The multimodal anesthetic-analgesic protocol was effective, with minimal signs of postoperative pain based on regular observations using a customized pain-assessment chart. Vital parameters remained largely within expected ranges, with minor deviations in respiratory rate and temperature. Injections missed the hypothalamic target by 0.5–2 mm, typically landing in the septal region due to interindividual anatomical variation.

**Conclusions:**

This study demonstrates the feasibility of stereotaxic neurosurgery in Svalbard rock ptarmigan by employing rigorous perioperative monitoring and a carefully tailored anesthetic-analgesic regimen. These results provide valuable guidance for future experimental procedures, improving both animal welfare and the reliability of neurosurgical research techniques in avian species.

## Background

The Svalbard rock ptarmigan (SRP, *Lagopus muta hyperborea*) is a subspecies of the Rock ptarmigan (*Lagopus muta*), a high-latitudinal, high-altitudinal grouse species. It permanently inhabits the Svalbard archipelago (74–81° north latitude), only migrates locally and is under Arctic conditions year-round [1, 2]. These birds have been shown to display photoperiod-dependent seasonal rhythms with many extraordinary adaptations to the High Arctic conditions, amongst other, marked seasonal changes in reproductive physiology, molting, appetite control and body mass [1, 3–8].

Due to its unique physiological characteristics, the SRP is an excellent model species for studies on photoperiodism along with circadian and circannual timekeeping [1, 4, 6, 8–10]. The limited understanding of the neuroendocrinological pathways responsible for the ability of birds to anticipate and respond appropriately to seasonal changes remains elusive, posing difficulty in developing timekeeping models for birds and, by extension, other species as well. An experimental assessment of seasonal rhythmicity in SRP could provide valuable insights into the plasticity of neural circuits controlling the anticipatory survival responses. This knowledge is crucial for evaluating the potential impacts of climatic disruptions on the health and resilience of organisms and ecosystems [11–13], especially in polar regions where climate change effects are most pronounced [14].

Stereotaxic neurosurgery has emerged as an important experimental technique for studying the neuroendocrinological underpinnings of circadian and circannual rhythms [15–17]. This method allows for precise targeting and manipulation of specific brain regions believed to be responsible for regulating timekeeping mechanisms. Performing stereotaxic neurosurgery, however, requires advanced surgical skills, meticulous anesthetic protocols and thorough analgesic management to ensure scientific accuracy and animal welfare. A precise understanding of species-specific physiology and anesthesia-analgesia requirements is therefore of utmost importance [18–21]. This presents a challenge in avian models due to their specialized anatomical and physiological features, interspecies variability, and a lack of protocols for anesthetics, analgesics, and other medications [22, 23].

Birds rely on a unique respiratory system with separate compartments for gas exchange and ventilation. The rigid lungs, minimally expandable and fixed to the thoracic walls, rely on air sacs to drive air flow through the parabronchi, where gas exchange occurs during inspiration and expiration [24–26]. Compared to mammals of similar size, birds have smaller functional lung reserve and larger tracheal dead space volume, rendering them more susceptible to hypoventilation and hypoxia under anesthesia [26, 27]. Birds also have proportionally larger hearts, higher cardiac index, and lower heart rates compared to mammals of similar body size, making them more sensitive to fluctuations in anesthetic depth, which can result in bradycardia or hypotension [23, 28, 29]. Birds are homoeothermic, with the cardiovascular system playing a major role in thermoregulation. While birds maintain a higher core body temperature than mammals of similar size, they are susceptible to hypothermia due to their higher surface area-to-body mass ratio [27–33].

Inhalant anesthetics are the preferred method for anesthetic induction and maintenance in birds with isoflurane and sevoflurane being the most common choices. They allow for rapid induction and recovery, and swift adjustments in anesthetic depth. Disadvantages include dose-dependent cardiopulmonary depression and hypotension [27, 28, 30, 32, 34]. Compared to mammals, birds are more sensitive to the effects of inhalant anesthetics as the parabronchial cross-current gas exchange is more efficient than the alveolar gas exchange [26, 35, 36]. On the other hand, without proper airflow through the parabronchi, efficient gas exchange can’t take place, and hypoxemia with hypercapnia develop rapidly [37].

Analgesia in birds should be pre-emptive and often requires a multimodal approach where medications with different pharmacological profiles and mechanisms can work synergistically to improve pain management. The use of opioids, non-steroidal anti-inflammatory drugs (NSAIDs), and local anesthetics reduces pain and inflammation, improves recovery and increases analgesic efficacy [27, 34]. Opioid analgesics used in birds are those that act on κ- and/or µ-opioid receptors, with butorphanol and buprenorphine being the most common in wildlife [27, 38]. Carprofen and meloxicam are the NSAIDs of choice [23]. Local anesthetics should be used primarily as an adjunct to general anesthesia to enhance pain prevention [33, 34].

Monitoring during anesthesia is essential and allows for adjustments in anesthetic depth and prompt response to critical events. At a minimum, heart and respiratory rates and rhythms should be monitored, focusing on individual trends rather than species-wide averages. Generally, these rates increase when the bird is experiencing pain and/or inadequate anesthesia and decrease with deep plane of anesthesia [23, 25, 27, 28, 32]. These parameters can also be altered by the presence of hypoxemia and/or hypercapnia [26, 29]. In species where such information is unavailable, allometric scaling can be used to predict resting heart and respiratory rates as a function of bodyweight [39–42].

Adaptations of the SRP to the climatic conditions of the High Arctic, which could provide additional challenges to anesthesia, include seasonally dependent presence of highly insulated plumage, substantial fat deposits (up to 35% of its body mass in winter) serving as insulation and energy storage [1, 43–47], and profound changes in metabolism [48]. Thus, in this species, it is imperative to consider its seasonal phenotype prior to planning any surgical procedures requiring general anesthesia. While birds under anesthesia are generally susceptible to hypothermia, in naturally cold-adapted avian species, hyperthermia might pose a bigger risk [49]. Moreover, the amount of accumulated fat tissue and associated metabolic changes might alter the overall pharmacokinetic and pharmacodynamic profile of administered medications [50–52].

Here, we present the results of a pilot study in which we injected a neuronal tracer into anatomically defined brain areas of SRP under anesthesia, with concurrent analgesia, thereby defining a feasible and efficient protocol for stereotaxic neurosurgery in this species. We also provide recommendations for further refinement of this protocol for future experimental work.

## Methods

### Animals

SRP (*n* = 6; 4 males, 2 females; Table 1) were either bred at UiT – The Arctic University of Norway from wild birds originally captured on the Svalbard archipelago (Birds 1, 2, 3, 4, and 6) or imported as chicks from the Svalbard archipelago and subsequently housed at UiT – The Arctic University of Norway (Bird 5). During the study period, the birds were housed in individual indoor cages (100 × 70 × 45 cm) and were visually but not acoustically separated. They were provided with food (Grouse pellets, Lundi) and water *ad libitum*, except for the 6 h fasting period preceding surgery. One ml/L of 5–6% apple cider vinegar (Eplecider-eddik, Emin) was added into drinking water to maintain healthy crop pH and digestion. All surgeries took place in May and June with birds expressing the appropriate seasonal phenotype (depletion of fat stores, molt from white winter plumage to brown summer plumage, increased activity, pre-breeding/territorial behavior [1, 46, 47]). During the first 24 h after surgery, birds were housed in an individual padded cage to prevent injuries.

**Table 1:** Demographic data for the Svalbard rock ptarmigan involved in this pilot study

Bird ID	Age	Body mass (g)	Sex
1	10 months	465	M
2	2 years 10 months	798	F
3	6 years 10 months	597	F
4	6 years 10 months	838	M
5	1 year 11 months	826	M
6	11 months	491	M

### Stereotaxic neurosurgery

**Fig. 1:**
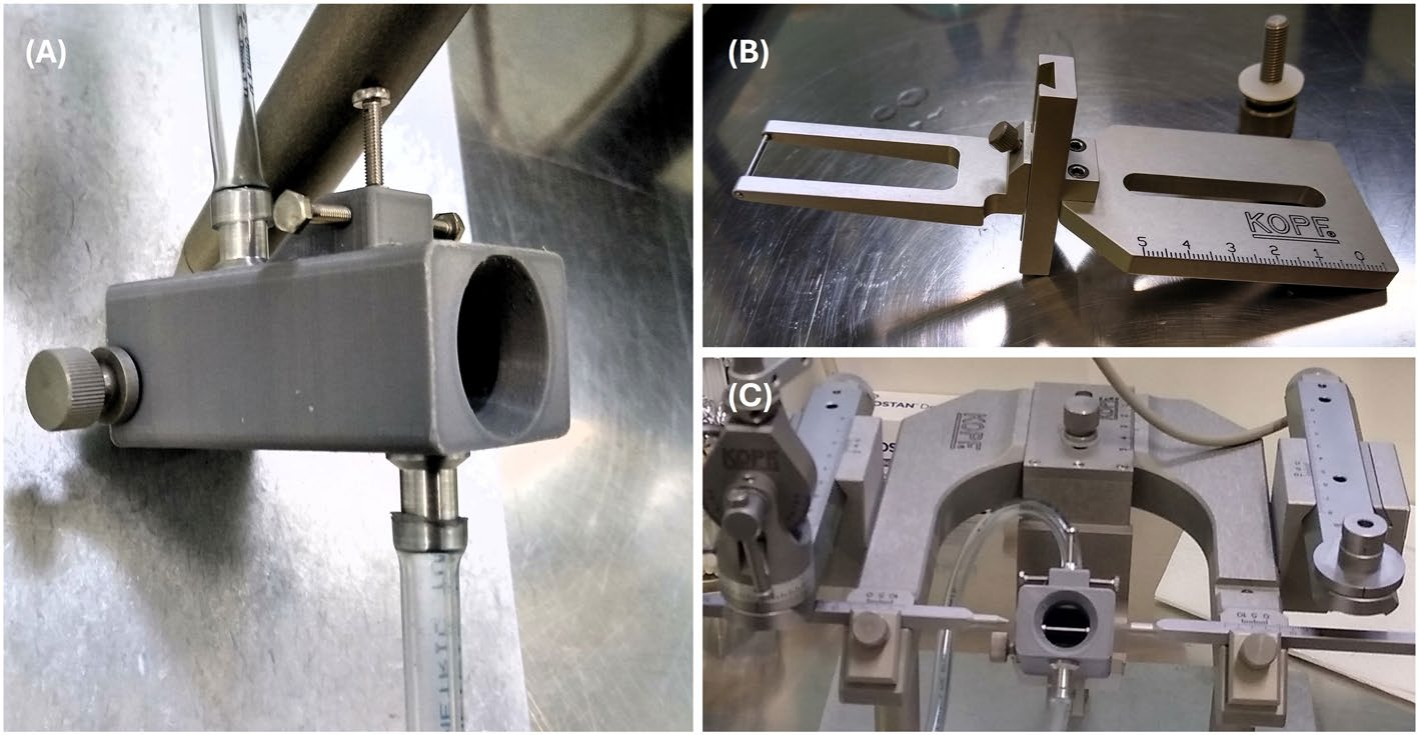
Custom 3D-printed anesthetic mask. **A** The anesthetic mask, modeled after a rat anesthetic mask (Model 906, Rat Anesthesia Mask, David Kopf Instruments), allows for insertion of the adaptor containing the beak bar to position and secure the head in the stereotaxic apparatus. Beak is clamped using the screws on top. There is an inlet and outlet port for gas delivery and removal, respectively. **B** The adaptor (Model 918, Pigeon Adaptor, David Kopf Instruments) including the beak bar (left) can easily slide into the anesthetic mask. **C **The anesthetic mask with the inserted adaptor (beak bar can be seen in the middle) mounted onto the stereotaxic instrument; non-rupture blunt-end ear bars can be seen on either side

After anesthetic induction, feathers on the top of the head and covering the external ear canals were removed with scissors. Birds were securely positioned into a stereotaxic apparatus (Model 902 Small Animal Stereotaxic Instrument, David Kopf Instruments) using a beak bar (Model 918 Pigeon Adaptor, David Kopf Instruments) in the mouth and non-rupture blunt-end ear bars (Model 955 Non-Rupture Ear Bars, David Kopf Instruments) in both ears (Fig. 1B, C). The scalp was disinfected (Betadine, Mylan; 100 mg/ml povidone iodine solution), and a skin incision was made with a scalpel blade to expose the cranium. A craniotomy (~ 2–4 mm in diameter) was performed with a sterile electric drill (Portable micromotor, Hager & Meisinger GmbH; Anchor Screw Drill Bits, Harvard Apparatus). After cutting the meninges, a 33 G blunt-end needle (33G Blunt NanoFil Needle, World Precision Instruments) filled with a neuronal tracer (biotin dextran amine, 10,000 MW; NeuroTrace™ BDA-10,000 Neuronal Tracer Kit, Invitrogen™) was inserted into the brain tissue and 0.5 µl of the tracer were delivered using a microsyringe pump (UltraMicroPump3, World Precision Instruments) with a flow rate of 100 nL/min. The coordinates for the injection site were based on a stereotaxic atlas designed for chicken [53], the closest related species with such an atlas available, with the target area being the anteromedial hypothalamic nucleus (Fig. 6A). The needle was kept in place for ~ 10 min, to ensure the tracer traveled from the injection site and was not pulled back through the injection channel. The injection was administered unilaterally. The exposed brain area was overlaid with a sterile surgical gelatin sponge (Spongostan Dental, Ethicon, Johnson&Johnson), covered with dental cement (Super-Bond Universal Kit, Technomedics Norge AS) and the skin was sutured using size 6 − 0 polyamid suture with C-2 reverse cutting needle (Ethilon, Ethicon, Johnson&Johnson).

### Intraoperative anesthetic and analgesic protocol

Prior to surgery, birds were fasted for 6 h [28]. Birds were induced with 3–5% isoflurane (Isofluran, Baxter) in 100% O_2_ [30, 34, 54] delivered via a vaporizer (U-1200 Anaesthesia Unit, Agntho’s AB) connected to an anesthetic mask. Maintenance of anesthesia was achieved with 1–3% isoflurane [32, 34, 54] in 80–100% O_2_ at a flow rate of 0.5–1 L/min delivered via a vaporizer connected to a custom-made 3D-printed anesthetic mask (Fig. 1A, C) allowing for use of the beak bar to securely and precisely position the head in the stereotaxic apparatus.

**Fig. 2:**
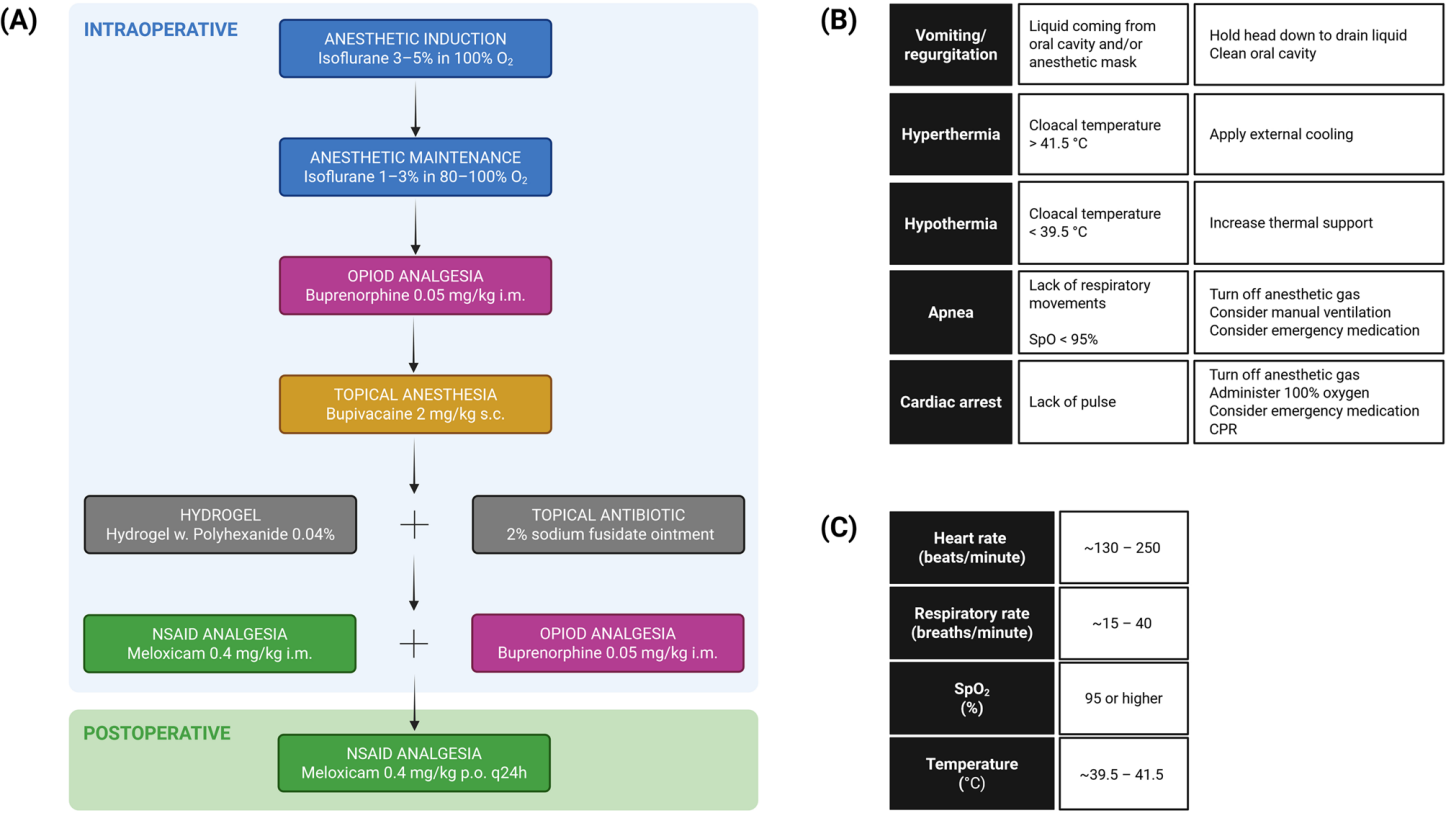
Schematic overview of the anesthetic-analgesic protocol in Svalbard rock ptarmigan. **A** The medications used intraoperatively (from induction of anesthesia to cessation of anesthesia; blue background) and in the postoperative period (green background). **B **Potential emergency situations, their symptoms and treatment options; adapted from [23, 32, 59]. **C** Recommended ranges for physiological parameters (heart rate, respiratory rate, oxygen saturation – SpO2, cloacal temperature) for Svalbard rock ptarmigan under general anesthesia. CAVE: Rather than using a published heart rate for a particular species, special attention needs to be paid to abrupt changes in rate/rhythm [27, 32]! Created in https://BioRender.com

When the depth of anesthesia was sufficient (absent pedal withdrawal and palpebral reflexes, muscular relaxation, loss of righting reflex [23, 27]), buprenorphine (Bupaq vet., VetViva Richter, 0.3 mg/ml injection solution) 0.05 mg/kg i.m [23, 30, 54, 55]. was administered into the pectoral muscle. After placing the bird into the stereotaxic apparatus, bupivacaine (Marcain, Aspen, 0.25% w/v injection solution) 2 mg/kg s.c [34, 54, 56, 57]. was injected at the incision site on the head. Eye ointment (Simplex Øyesalve, Teva) was applied to the eyes to prevent eye dryness [34, 58]. At the end of the surgery, while still under anesthesia, antibiotic ointment (Fucidin, LEO, 2% sodium fusidate) and hydrogel (HydroGel w. Polyhexanide 0.04%, KRUUSE) were applied to the sutured wound to support healing. In the final analgesic regime, after adjustments (see *“Results”*), a second dose of buprenorphine 0.05 mg/kg i.m. and meloxicam (Metacam vet., Boehringer Ingelheim Vetmedica GmbH, 5 mg/ml injection solution) 0.4 mg/kg i.m [23]. were administered into the pectoral muscle prior to discontinuation of inhalant anesthesia. The pharmacological protocol is summarized in Fig. 2A.

We were prepared to address emergency situations (e.g., hypothermia/hyperthermia, apnea, cardiac arrest, regurgitation) by devising an emergency protocol summarized in Fig. 2B. Heating pad (Homeothermic Monitoring System, Harvard Apparatus) was used if the cloacal temperature dropped below 39.5 °C. In case the cloacal temperature rose above 42 °C, we were ready to apply external cooling with ice packs. Emergency medication was readily available during all surgeries performed (Epinephrine (1:1000) 0.5–1.5 ml/kg i.m., Doxapram 20 mg/kg i.m or intranasal, Naloxone 2 mg/kg i.v [23, 32, 54, 56, 57, 59]).

### Intraoperative monitoring of physiological parameters

We had continuous monitoring throughout the surgeries. Heart rate and oxygen saturation were monitored via a pulse oximeter (Covidien Nellcor PM10) with the sensor placed on the foot [27, 32]. Respiratory rate was assessed by observing the frequency of respiratory movements and listening to potential respiratory noises. The core body temperature was monitored with a thermometer with a cloacal probe (Homeothermic Monitoring System, Harvard Apparatus). The plane of anesthesia was reassessed prior to any potentially painful intervention.

### Postoperative period and monitoring

Postoperatively, birds were monitored using pulse oximeter, thermometer, and visual observation until awakening, but the pulse oximeter sensor and thermometer probe were removed before full consciousness to prevent injuries. After awakening, the birds were moved into a padded cage and observation continued until fully awake and standing [60]. Birds were then monitored at 3, 6 and 24 h after surgery and then at least q12h until sampling using a customized pain assessment chart (Table 2). After 24 h, the birds were moved from the padded cage into their regular cage. Meloxicam (Metacam vet., Boehringer Ingelheim Vetmedica GmbH, 1.5 mg/ml oral suspension) 0.4 mg/kg p.o [23]. was provided q24h administered with lingonberries (*Vaccinium vitis-idaea*) to facilitate voluntary intake, and administration continued until euthanasia (Fig. 2A). Generally, appropriate measures – i.e., consulting the attending veterinarian, increasing the frequency of monitoring, or providing additional analgesia – were implemented when clinical signs listed in Table 2 did not show gradual improvement within the first 24 h post-operatively, or reoccurred thereafter.

**Table 2:** Pain assessment chart for postoperative monitoring in Svalbard rock ptarmigan

Surgical wound	*Swelling*
	*Redness*
	*Warmth*
	*Discharge*
Breathing	*Wheezing or other abnormal noise*
	*Rapid and/or labored breathing*
Demeanor, posture, and appearance	*Low activity*
	*Hunched appearance*
	*Fluffed up feathers*
	*Closing eyes*
Locomotion	*Slower speed*
	*Difficulty moving*
	*Falling*,* stumbling*
	*Immobility*
Temperament and behavior	*Aggression or passivity depending on normal behavior*
	*Lethargy*,* apathy*
	*Decreased interest in surroundings*
	*Escape reactions*
	*Feather-destructive behavior and/or self-mutilation*
Eating and/or drinking habits	*Reduced food and water intake*
	*No interest in food and/or water*
Feces	*Any change in color*,* consistency*,* amount*,* or odor*
Vocalizations	*Birds that are normally vocal are quiet*,* or vice versa*
	*Vocalization on palpation of the incision site*

Adapted from [22, 23, 27, 126]

Birds were euthanized 7 days post-surgery with an anesthetic overdose of pentobarbital (Exagon vet., VetViva Richter; 400 mg/ml pentobarbital sodium, 1–2 ml/kg, intraperitoneal injection) preceded by anesthetic induction with 4% isoflurane.

### Tissue processing for verification of injection site

Briefly, birds were transcardially perfused with saline containing 20 IU/ml heparin [61] and subsequently 4% PFA in 1X PBS. Whole brains were dissected and placed in a post-fixative solution, 4% PFA in 1X PBS, overnight at 4 °C. Then, brains were washed in 1X PBS and cryoprotected in a 30% sucrose solution. Brains were sectioned at −20 °C on a cryostat (Leica CM3050 S) at 40 μm sectioning thickness. Free-floating sections were stored in a cryoprotective solution (30% sucrose dissolved in 40% 1X TBS + 30% glycerol + 30% ethylene glycol) at −20 °C until further processing. Brain sections were stained using avidin-horseradish peroxidase (avidin-HRP; NeuroTrace™ BDA-10,000 Neuronal Tracer Kit, Invitrogen™) and diaminobenzidine (DAB; NeuroTrace™ BDA-10,000 Neuronal Tracer Kit, Invitrogen™). Sections were mounted on microscope slides (SuperFrost Plus GOLD, Epredia™) and scanned with a brightfield microscope (Olympus VS120).

### Data analysis and visualization

Four monitored physiological parameters were analyzed using descriptive statistics: heart rate, respiratory rate, oxygen saturation and cloacal temperature. The time course and intraoperative stability of these parameters were evaluated, along with interindividual variation and possible fluctuations due to pharmacological effects and/or painful stimuli. Data points used for statistical analyses are distributed approx. 2–5 min apart. Similarly, as per a study performed in pigeons (*Columba livia*) [62], we defined a time window with the highest potential for painful stimuli (further referred to as “painful window”) from when the craniotomy drilling started to when the needle was inserted into the brain tissue. We then compared the values within this window against all other values using a paired two-tailed t-test. Additionally, allometric scaling was performed for heart rate: f_h_ = 155.8 x M^−0.23^ and respiratory rate: f_r_ = 17.2 x M^−0.31^, where M represent bodyweight in kg [39–42]. Statistical tests were implemented in GraphPad Prism v10.5.0. QuPath v0.5.0 was used for visualization and assessment of microscopy images.

## Results

### Anesthetic and analgesic protocol

The initial analgesic protocol, used only in Bird 1, consisted of a single dose of buprenorphine 0.05 mg/kg i.m. administered at the start of the surgery, followed by meloxicam 0.2 mg/kg i.m [63]. given at the end of the surgery. This dosing regimen didn’t provide adequate perioperative analgesia. Bird 1 showed signs of acute pain during recovery, including reduced mobility and interest in surroundings, feather erection, closed eyes, tremor, diarrhea and little interest in food during the first 48 h post-surgery. Consequently, Bird 1 received another dose of buprenorphine 0.05 mg/kg i.m. 24 h post-surgery, and meloxicam 0.4 mg/kg i.m. q24h until he started eating again, after which meloxicam 0.4 mg/kg was provided p.o. q24h. All further surgeries were postponed while the pain management protocol was reassessed.

For subsequent surgeries (Birds 2, 3, 4 and 6), we included buprenorphine 0.05 mg/kg i.m. both at the start and at the end of the surgical procedure, and meloxicam 0.4 mg/kg i.m. given at the end of the surgery and thereafter 0.4 mg/kg p.o. q24h (Fig. 2A). This altered pain management protocol showed good results with birds recovering quickly and not showing behavioral signs of pain. They all started eating within 6 h after regaining consciousness and had normal food intake during the postoperative period. Based on the pain assessment chart (Table 2), birds displayed low activity and apathetic behavior immediately following surgery but gradually regained normal activity levels within 24 h.

Bird 5 died unexpectedly during anesthetic induction prior to commencing the surgical procedure. The sudden cardio-pulmonary arrest manifested as abrupt cessation of respiration and heartbeat, muscle rigidity and piloerection of feathers. As this happened before any monitoring had begun, we do not have any records of physiological functions. We immediately turned off the anesthetic gas supply, started oxygenation with 100% oxygen and attempted cardio-pulmonary resuscitation.

Emergency mediation was not required at any point during any of the surgical procedures.

The entire surgical procedure, from first skin incision to finished sutures, lasted 71.4 ± 9.7 min (mean ± SD rounded up to one decimal point). The duration of anesthesia from induction to turning off the anesthetic gas was 119.6 ± 14.7 min (Table 3).

**Table 3:** Monitoring of selected physiological parameters in Svalbard rock ptarmigan undergoing stereotaxic neurosurgery

Bird ID	HR (beats/min)	HR – scaled (f_h_) (beats/min)	RR (breaths/min)	RR – scaled (f_*r*_) (beats/min)	SpO_2_ (%)	Cloacal temperature (°C)	Anesthesia duration (min)	Surgery duration (min)
1	187.0 ± 44.3	185.8	21.6 ± 6.2	21.8	98.9 ± 1.0	40.1 ± 0.3	134	73
2	149.5 ± 14.8	164.1	38.0 ± 6.5	18.4	98.1 ± 1.3	39.5 ± 0.1	116	85
3	152.8 ± 6.5	175.4	24.8 ± 4.6	20.2	100.0 ± 0	39.6 ± 0.3	120	75
4	224.6 ± 23.2	162.3	39.0 ± 4.0	18.2	96.4 ± 1.3	39.5 ± 0.2	131	63
6	205.5 ± 13.9	183.5	18.2 ± 4.4	21.4	100.0 ± 0.2	39.1 ± 0.2	97	61

Values represent mean ± SD rounded up to one decimal point. Bird 5 underwent sudden cardiorespiratory arrest during the induction of anesthesia before monitoring could have begun. Anesthesia duration represents time from induction to turning off anesthetic gas. Surgery duration represents time from the first skin incision to the last suture. HR – heart rate, RR – respiratory rate. Scaled values for heart rate (f_h_) and respiratory rate (f_r_) are calculated based on the following allometric scaling equations: f_h_ = 155.8 x M^−0.23^, f_r_ = 17.2 x M^−0.31^, where M represent bodyweight in kg [39–42]

### Physiological parameters

**Fig. 3:**
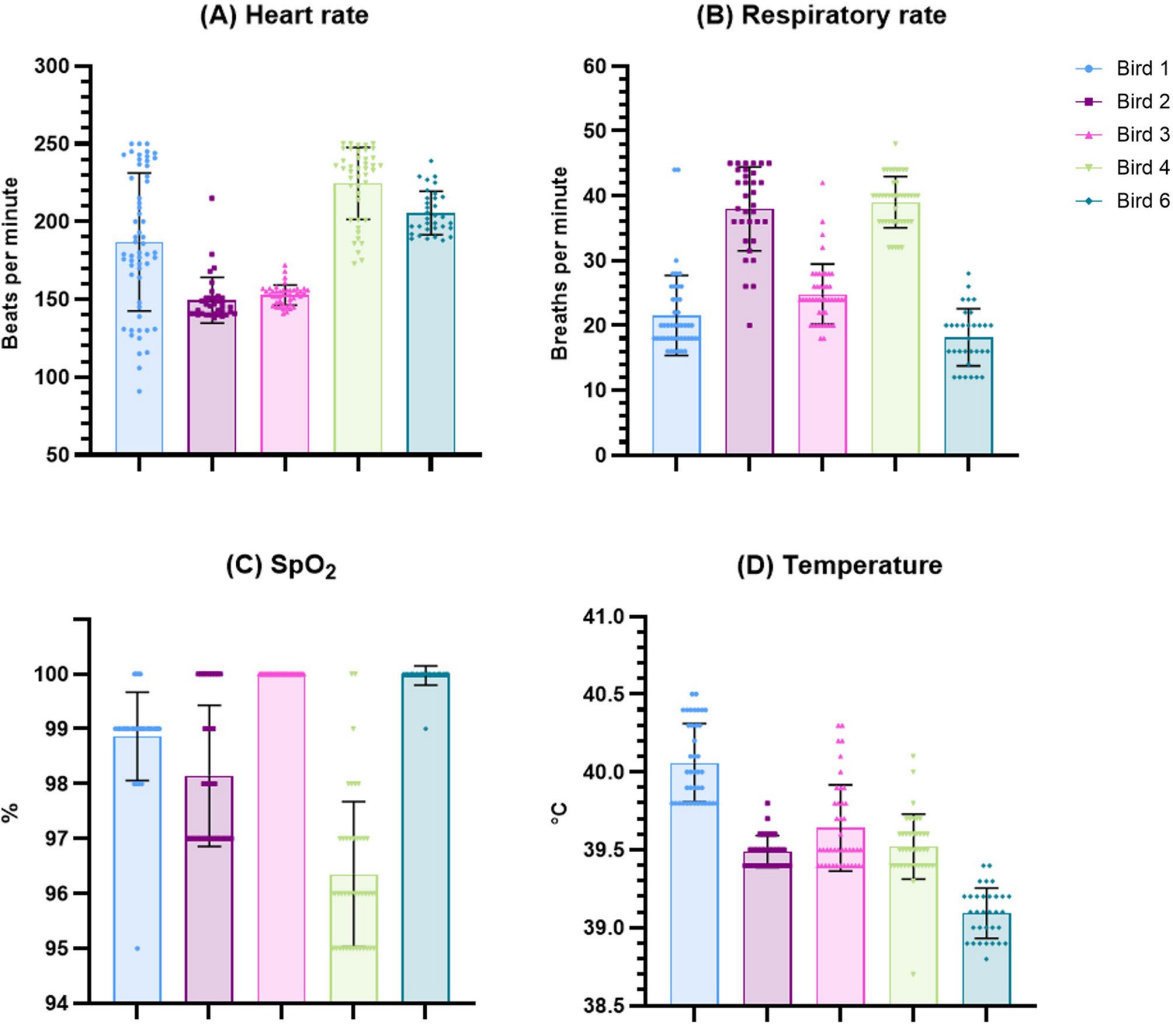
Mean values of measured physiological parameters. Results are displayed as mean with error bars depicting ± SD. (**A**) Heart rate for individual birds in beats/min. (**B**) Respiratory rate for individual birds in breaths/min. (**C**) Oxygen saturation for individual birds in %. (**D**) Cloacal temperature for individual birds in °C

**Fig. 4:**
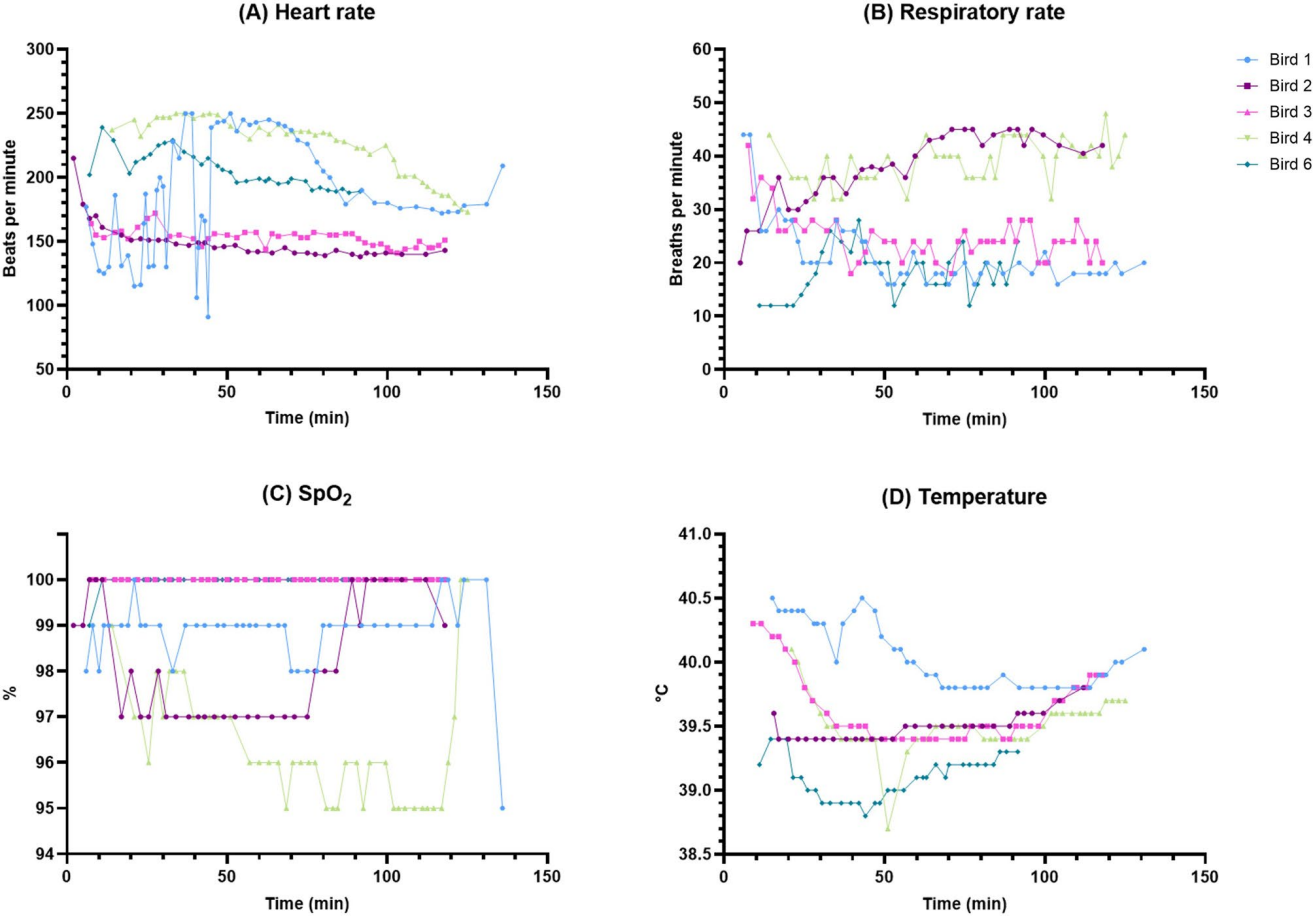
Time course of measured physiological parameters. (**A**) Heart rate for individual birds in beats/min. (**B**) Respiratory rate for individual birds in breaths/min. (**C**) Oxygen saturation for individual birds in %. (**D**) Cloacal temperature for individual birds in °C

The values of monitored physiological parameters for operated birds are summarized in Table 3; Fig. 3, shown as mean ± standard deviation (SD). The mean values for heart rate and oxygen saturation fell within the expected ranges based on literature [64–67]. Respiratory rates were slightly higher than the expected range of 10–30 breaths/min [64, 65] with mean values of 38 and 39 for Bird 2 and Bird 4, respectively. Temperatures were slightly lower than anticipated [6, 65, 68] with mean values of 39.5 and 39.1 for Bird 2 and Bird 6, respectively. Cloacal temperature didn’t rise over 42 °C at any point during any of the surgeries. All 5 birds that underwent the complete surgical procedure recovered well and survived the 7-day postoperative period until time of sampling. Recommended ranges for heart rate, respiratory rate, oxygen saturation and core body temperature in SRP under general anesthesia, taking into account the results of this study, are summarized in Fig. 2C. We assessed the time course of the monitored physiological parameters and their fluctuations and stability throughout the surgical procedure. Heart rates were stable in Birds 2, 3, 4 and 6; Bird 1 showed substantial fluctuations in heart rate during the first ~ 50 min after induction before stabilizing (Fig. 4A). Respiratory rate seemed to fluctuate during the first ~ 30 min after induction (increasing in Bird 2 and 6, decreasing in Bird 1, 3 and 4) but remained stabilized for the rest of the procedure with overall higher values in Bird 2 and 4 (Fig. 4B). Oxygen saturation was stable throughout surgery and didn’t drop below 95% at any point for any of the birds (Fig. 4C). We observed a gradual decline in cloacal temperature over the first ~ 50 min after induction, which was successfully counteracted by using a heating pad once temperatures dropped to 39.5 °C (Fig. 4D). Only Bird 6 maintained a mean cloacal temperature of 39.1 °C despite the use of a heating pad (Table 3; Fig. 3D).

**Fig. 5:**
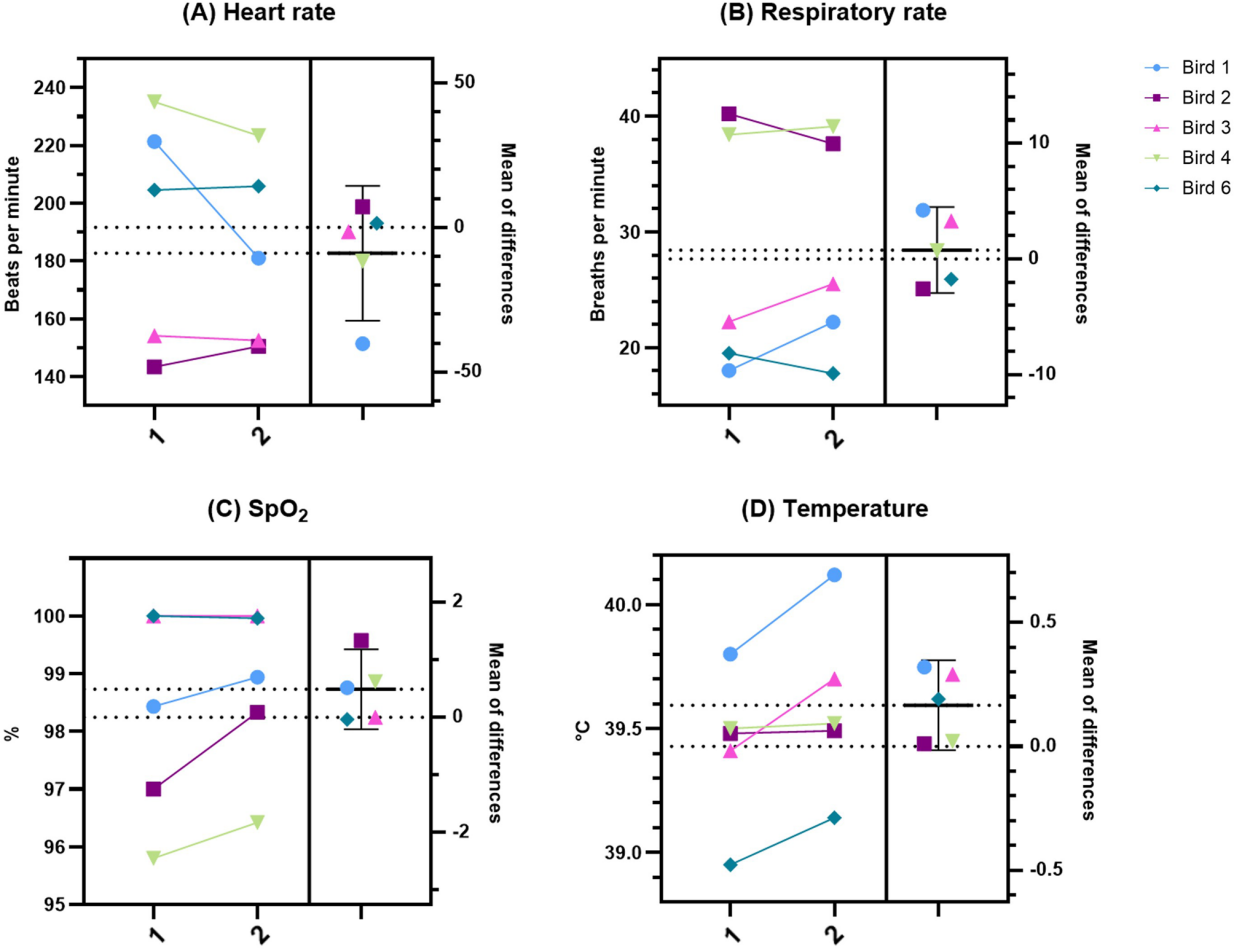
Estimation plots of measured physiological parameters showing comparison between the window with the highest potential of painful stimuli and the rest of the procedure. Column 1 shows means of values that fall between the beginning of craniotomy drilling and needle insertion into brain tissue. Column 2 shows means of all other values. The left y-axis shows the data; the right y-axis shows the difference between means and the calculated effect size as a 95% confidence interval. Y=0 on the right axis is aligned at the position of the mean of Column 1 plotted on the left axis. (**A**) Heart rate for individual birds in beats/min. (**B**) Respiratory rate for individual birds in breaths/min. (**C**) Oxygen saturation for individual birds in %. (**D**) Cloacal temperature for individual birds in °C

When comparing values of monitored physiological parameters in the painful window (Bird 1: 66:30–84:30, Bird 2: 50:20–70:00, Bird 3: 54:30–79:15, Bird 4: 66:49–77:45, Bird 6: 42:17–59:36; *Bird ID: min: sec–min: sec*) against all other values (Fig. 5), no statistically significant results were discovered (Heart rate: *p* = 0.3431, t = 1.075, df = 4; Respiratory rate: *p* = 0.5947, t = 0.5772, df = 4; SpO_2_: *p* = 0.1243, t = 1.941, df = 4; Temperature: *p* = 0.0639, t = 2.542, df = 4).

### Injection site

**Fig. 6:**
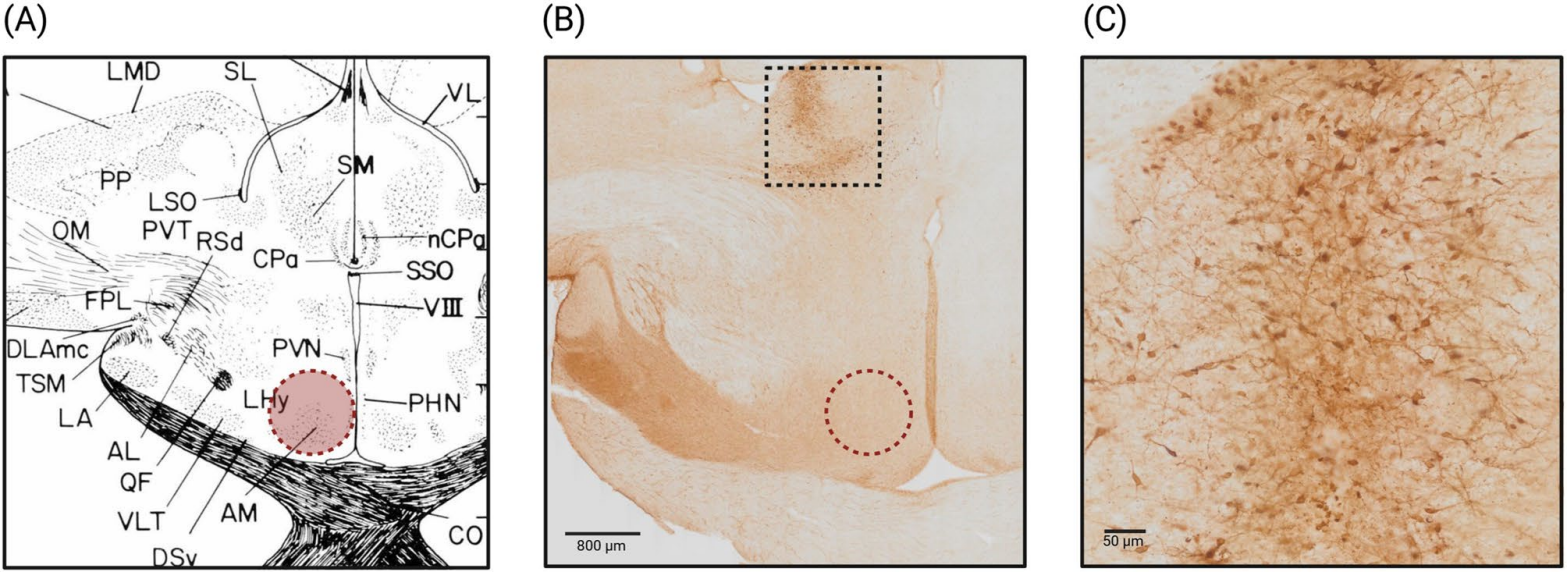
Example of injection site visualization. **A** Target anatomical plane from A Stereotaxic Atlas of the Brain of the Chick (Gallus domesticus) [53]. The target area, anteromedial hypothalamic nucleus, is marked with a dashed dark red circle. **B** DAB-stained brain section with the injection site localized to the septal region, marked with dashed black square. Dashed dark red circle, again, marks the intended target site. **C** Detailed view of the BDA-positive neurons in the injection site. Created in https://BioRender.com

While our target site was the anteromedial hypothalamic nucleus (Fig. 6A), the injection site in all five birds was instead localized to the septal region or an area closely adjacent. Overall, assessing the injection placement in all birds, the injection site was ~ 1–2 mm ventral, ~ 1 mm anterior and ~ 0.5–1 mm lateral from reaching the intended target site (Fig. 6B). Cellular uptake of the neuronal tracer was confirmed along with its distribution throughout the neuronal processes (Fig. 6C).

## Discussion

We aimed at developing a protocol for anesthetic and analgesic management of Svalbard rock ptarmigan undergoing stereotaxic neurosurgery with the purpose of delivering neuronal tracer injections to defined brain areas hypothesized to control seasonal timekeeping in avian species. We successfully performed 5 surgeries and devised a protocol for perioperative care prioritizing animal welfare. This pilot study represents the very first time such surgery was performed in this species, and its results constitute a useful resource for further improvement of avian anesthesia, especially under research conditions.

Isoflurane was used for anesthetic induction and maintenance due to our research group’s positive experience with using isoflurane in SRP [6, 63] and its successful use in other avian species undergoing comparable surgical procedures [17, 69–77]. Inhalation anesthetics were also preferred over injectable agents to minimize handling-induced distress and injection-associated pain. Manual restraint, particularly in cold-adapted species like the SRP, can lead to hyperthermia, myopathy, and cardiac arrhythmias [23, 28, 32, 78]. Although injectable agents, such as the ketamine/xylazine mixture, have been successfully used in various avian species [79–84], they present several challenges such as the inability to quickly modify the effects of the drug after it has been administered, cardiopulmonary depression, prolonged and violent recoveries, difficulties in administering precise volumes and species-specific variations in response [23, 33].

Isoflurane proved effective in keeping SRP in surgical plane of anesthesia, but its overall safety in this species warrants further investigation, especially considering its potentially harmful cardiac side effects. Isoflurane has a documented potential to cause cardiac arrhythmias, as reported in bald eagles (*Haliaeetus leucocephalus*) where isoflurane caused periods of apnea, tachycardia, hypertension and sudden death [85, 86]. Also, in cinereous vulture (*Aegypius monachus*), increasing isoflurane concentrations during spontaneous ventilation led to dose-dependent increase in heart rate and decrease in arterial blood pressure with one bird developing sinus arrhythmia [87]. In chickens, on the other hand, although isoflurane produced dose-dependent depression of arterial blood pressure, heart rate remained unchanged over a range of 1–2 minimal anesthetic concentration [88]. In this study, Bird 1 displayed substantial fluctuations in heart rate during the first ~ 50 min after induction; equipment malfunction or human error with the pulse oximeter sensor cannot be excluded but are unlikely due to careful assessment of the ability of the pulse oximeter to provide stable readings prior to and throughout the procedure. As the stroke volume can vary beat-to-beat, arrhythmias should be considered a likely cause of heart rate fluctuations detected via pulse oximetry. For further studies, ECG monitoring is highly recommended to identify potential arrhythmogenic effects of isoflurane in avian anesthesia. Bird 5 underwent sudden cardiorespiratory arrest during induction, prior to administration of any medication other than isoflurane. Without ECG monitoring, we could not confirm the presence of arrhythmias and were only able to rely on data from the pulse oximeter.

A potential alternative to isoflurane is sevoflurane. In several avian species, the use of sevoflurane compared to isoflurane resulted in faster induction and/or recovery due to decreased blood and tissue solubility [32, 58, 85, 89, 90]. In bald eagles [85] and pigeons [90], sevoflurane was found to be associated with fewer adverse cardiac effects. Additionally, sevoflurane is less irritating during mask induction than isoflurane [28].

Another explanation for the adverse cardiac symptoms in Bird 1 and 5 could be a handling-induced stress response. In birds that are restrained, heart rate can be up to 401% higher than that of birds at rest [91]. In streaked shearwater *(Calonectris leucomelas)*, handling induced a strong stress response, exhibited by an elevation in heart rate from 160 to nearly 300 beats/min, which required up to 2 h to recover to its resting state [92]. In chickens, handling was documented to cause a rise in mean heart rate of 51 beats/min accompanied by rise in mean core body temperature of 0.5 °C [93]. This underlines the importance of minimizing handling while working with avian species, especially wild species such as the SRP.

Inhalant anesthesia-related mortality in birds is reported to be up to 3.4% [94], 3.88% [95], and 4.45% [96], respectively. The majority of deaths seem to occur in the post-anesthesia phase with less than 19% of anesthesia-related deaths occurring during the induction and maintenance phases [96]. A study focusing on the occurrence of cardiopulmonary arrest in avian species reports that 63.4% of birds experienced cardiopulmonary arrest in relation to anesthesia or sedation [97]. In our study, we saw one anesthesia-related death of Bird 5 during the induction phase. The other 5 birds experienced uneventful induction and maintenance, achieved a stable surgical plane of anesthesia, recovered well and survived the postoperative period. While maintenance with an inhalant anesthetic appears to be a suitable choice for stereotaxic neurosurgery in SRP, the induction technique ought to be reassessed for prospective studies. We opted for induction via an anesthetic mask without premedication as most drugs used in premedication require i.m. injection and the associated handling and restraint may be as stressful as the induction alone [23, 98]. However, given that the observed adverse cardiac effects in Bird 1 and 5 might be attributable to the handling- and restraint-induced stress response, the use of premedication might be beneficial in this case. Premedication reduces fear, stress, and anxiety, makes for a smoother induction to general anesthesia and reduces the amount of inhalant anesthetics required for induction and maintenance with a consequent dose-dependent reduction in negative cardiovascular side effects (arrhythmogenicity, hypotension) [28, 30]. Furthermore, intranasal administration of sedatives, such as midazolam, has proven effective in birds [99–102] and can be an option in situations where i.m. injections are unpreferable.

The requirement of using a beak bar to secure the head in the stereotaxic apparatus can pose challenges to the maintenance of anesthesia via anesthetic gas. While many studies involving a comparable surgical procedure in avian species didn’t comment of their technique of maintaining inhalant anesthesia, several others report the use of “facemask” [17], “anesthesia mask” [71], “nose cone” [69], or “beak mask” [77]. Intubation is also feasible and was previously performed in a similar procedure in pigeons [81]. However, the use of the beak bar means that an endotracheal tube with smaller internal diameter must be used which can impose significant resistance to air flow [24, 98]. The head is further stabilized in the stereotaxic apparatus by clamping the beak, which could further compress the endotracheal tube [103]. Thus, in this study, we considered it more practical to maintain our birds via an anesthetic mask. On the other hand, endotracheal intubation offers the ability to provide manual ventilation, better control of anesthetic depth, prevention of aspiration and reduction of dead space volume [28, 98] and needs be considered for future studies especially with regard to emergency situations.

Pulse oximetry has been claimed to be unreliable in avian species due to differences in hemoglobin absorption characteristics between birds and mammals [32, 104–106]. Without formal validation – i.e., comparison with arterial blood samples across a range of oxygen saturations – the SpO₂ values cannot be considered quantitatively accurate. However, the blood of pigeons [106] and ducks (*Cairina moschata*) [107] was previously found to share similarities with the oxygen-hemoglobin dissociation curve of mammals, encouraging the role for pulse oximetry in avian anesthetic monitoring. In this study, SpO_2_ levels did not drop below 95% in any of the birds. While the data needs to be interpreted with caution, and heart rate is the only parameter from the pulse oximeter that can be deemed reliable, we consider it an important non-invasive real-time measurement variable which can serve as an indicator of any abrupt changes in the bird’s physiological status when assessed in full context of overall clinical state.

The anesthesia-related temperature decline in SRP aligns with previous studies in birds [65, 89, 108–110] and shows that the use of heating devices is warranted even in a cold-adapted Arctic species. In Bird 1, 3 and 6 the mean cloacal temperature was lower during the painful window compared to all other time points; this was likely caused by the anesthesia-induced decrease in thermoregulatory response combined with the use of a heating pad to counteract this effect (Fig. 5D). In Bird 1, there could be a link between adverse cardiac symptoms and the overall higher mean temperature levels, as this was described previously following a stress-response in chicken [93]. Nonetheless, all events between craniotomy drilling and needle insertion happened after the heart rate and temperature became stable (66:30–84:30) and there was no observable direct response to any of the potentially painful stimuli.

Allometric scaling can be used to calculate resting heart and respiratory rates in species where such information is unavailable [39–42]. Here, we compared allometrically scaled values against empirically measured values (Table 3). While the mathematically predicted values offered a good general estimate, they often differed from the measured values thus highlighting the limitations of allometric scaling determined as a function of bodyweight and the importance of taking into account species-specific characteristics along with interindividual variability.

Guidelines for fasting vary greatly and depend on the size, species and physiological status [27, 28, 30, 32]. Since birds have a high metabolic rate and relatively poor hepatic glycogen stores compared with mammals, there is a higher risk of hypoglycemia when prolonged periods of fasting are imposed and it is therefore also important that birds eat soon after recovery from anesthesia [28]. On the other hand, a brief excitatory stage may occur while birds are recovering from anesthesia, which may be accompanied by regurgitation; water and feed should therefore only be offered after the bird has regained full consciousness [23, 27, 32]. We didn’t experience any of these issues during surgery or the postoperative period and thus consider the fasting period of 6 h appropriate for SRP.

Opioids are generally safe in birds, with dose-dependent adverse effects and variable analgesic response between avian species [38]. Some of this variability is due to differences in the proportion and localization of opioid receptor subtypes (κ, µ, δ) in the central nervous system [111–114]. Unfortunately, there is no published information on the distribution of opioid receptors in the central nervous system of the SRP. In domestic chickens, another Galliform species, µ-receptors were found to be predominant over κ-receptors in the forebrain and midbrain [111]. Common opioids in avian practice include tramadol, hydromorphone, fentanyl, butorphanol and buprenorphine with these agents differing in their receptor binding affinities [38].

Buprenorphine is believed to be a mixed agonist/antagonist with a complex pharmacological profile acting as a partial agonist at µ-opioid receptors and as an antagonist at κ- and δ-opioid receptors. Its analgesic effect is believed to stem mainly from µ-opioid receptor agonism [38, 115–119]. Buprenorphine has unusual receptor-binding characteristics – it binds strongly to and dissociates slowly from opioid receptors and has a long-acting analgesic effect in mammals; hence, plasma buprenorphine concentration may decline rapidly while its analgesic effect remains [38, 120, 121]. Results of pharmacokinetic and pharmacodynamic studies on buprenorphine in birds differ greatly between species and even sexes [38]. Buprenorphine was chosen as the opioid component of the multimodal analgesic approach due to its unique pharmacokinetic and pharmacodynamic properties, offering prolonged analgesia despite rapid plasma clearance. Its sustained effect was considered essential for effective pain management throughout and after the procedure. The dose of 0.05 mg/kg i.m. was previously successfully used in a comparable procedure in European barn owls (*Tyto alba*) [82]. Our initial opioid regimen consisted of a single dose of buprenorphine (0.05 mg/kg i.m.) at the start of surgery; this proved inadequate, however, as Bird 1 exhibited signs of acute pain during recovery. Following protocol reassessment, subsequent birds received buprenorphine (0.05 mg/kg i.m.) both at the start and end of surgery, which resulted in improved pain control and faster recovery. While we are satisfied with the effects of buprenorphine, a better knowledge of the distribution and proportion of opioid receptors in the central nervous system of SRP would help further improve the analgesic regimen for this species.

NSAIDs work by decreasing the production of prostaglandins, which promote inflammation, pain and fever [23, 27]. They may, however, cause renal failure and the likelihood of this varies between avian families. NSAIDs should therefore be used with care if a dose is extrapolated from one avian species to another [23]. Initially, we chose a dosage of 0.2 mg/kg meloxicam based on our previous experience with SRP undergoing surgery [63]. This dose proved to be insufficient for this procedure, likely due to the pain associated with performing a craniotomy, and the dosage was increased to 0.4 mg/kg [23].

Local anesthetics block ion channels to prevent pain impulse transmission, and should be combined with general anesthetics; they should not be used in awake birds due to the stress associated with handling and restraint [33, 34]. Bupivacaine, although conservatively used in birds due to concerns for toxicity [56, 122], remains one of the most clinically useful perioperative local anesthetic in mammals due to its long-lasting effect [123]. We opted for the use of bupivacaine over other local anesthetic agents based on literature search [56, 57] and data from other related species, mostly chicken [122, 124] and ducks [125].

The identification of reliable indicators of pain in avian species presents a substantial challenge, as reflected in our experience caring for the SRP. Pain indicators are often subtle and difficult to detect without specialized training, thus often rendering pain scoring systems unreliable (pers. obs. Kalinová and Thorvaldsen). The clinical state needs to be assessed in all its complexity, and it is imperative to understand each individual as well as the species [126]. Changes in behavior can serve as a potential sign of pain, their recognition, however, requires in depth knowledge of each individual bird, which can be particularly challenging to achieve in experimental settings. Some signs of pain, such as fluffed up appearance, reduced food intake or diarrhea are more obvious and easier to assess. Nonetheless, prior knowledge of baseline behavior is invaluable for detecting changes during the postoperative period and we recommend gathering this information ahead of commencing any potentially painful procedure. Bird 1 displayed multiple signs of acute pain immediately following surgery, including reduced mobility, behavior change, feather erection, tremor, diarrhea and lack of interest in food. This behavior was detected early due to careful monitoring and use of the pain assessment chart (Table 2). Adequate analgesia was provided for Bird 1, whose clinical state improved, and the analgesia regime was altered for the remaining birds.

The revised multimodal analgesic protocol, combining an opioid, an NSAID, and a local anesthetic proved highly effective for perioperative pain management in SRP. Birds 2, 3, 4 and 6 responded well to the altered pharmacological pain management regimen and only displayed low activity and apathetic behavior immediately following surgery. These signs could be attributed to slow recovery from anesthesia and/or the effect of buprenorphine given at the conclusion of surgery; in birds, the most common adverse effect of opioid use is sedation [38].

We noted substantial interindividual variability in skull size and shape which was reflected in anatomical parameters of the brain. This rendered targeting the designated brain area challenging as the area of interest is rather small and located deep within the hypothalamus. The fact that we didn’t manage to deliver the neuronal tracer into the intended target brain region highlights the species-specific anatomical differences. As there is no Rock ptarmigan stereotaxic brain atlas available, we opted to use an atlas developed for chickens [53]. This atlas was prepared using male broiler chicks two weeks of age. In our study, we had a highly heterogeneous group of birds, in terms of sex (4 males, 2 females), age (10 months – 6 years, 10 months) and body mass (465–838 g). This heterogeneity, combined with probable species-specific neuroanatomical variations, caused challenges in defining the coordinates for the injection site, resulting in the tracer being delivered into a brain area adjacent to the target site (Fig. 6B). The atlas’ authors declare 80% accuracy but point to a more realistic expectation of 50% accuracy when initially using this atlas [53]. For future experimental work involving stereotaxic neurosurgery in Svalbard rock ptarmigan, we recommend either (1) a homogenous group of birds with body and brain mass analogous to the birds used for development of the chicken brain atlas [53] or, (2) developing a new species-specific stereotaxic brain atlas.

Important limitations of this study include the lack of i.v. access and continuous ECG monitoring throughout the surgical procedure. Established i.v. access could have allowed timely pharmacologic intervention in emergency situations, while the presence of ECG could have enabled prompt identification and correction of heart rate fluctuations [97]. Whilst i.m. administration of emergency medication was part of the emergency protocol in this pilot study, current resuscitation guidelines in veterinary emergency and critical care recommend i.v. or i.o. administration as the preferred route for emergency drug delivery [97, 127]. Thus, for prospective studies, we argue for establishing i.v. line and ECG monitoring as a standard part of the monitoring protocol to ensure adherence to best practices in peri-anesthetic clinical veterinary care and to improve the ability to respond effectively to life-threatening complications.

## Conclusions

In conclusion, this pilot study presents a feasible and efficient protocol for stereotaxic neurosurgery and perioperative management of Svalbard rock ptarmigan. Nonetheless, further refinement is needed to enhance the safety of the proposed protocol, including the use of premedication and the availability of intubation, manual ventilation, i.v. access and continuous ECG monitoring. Considering the lack of well-established anesthetic-analgesic protocols in avian species, the novelty of this technique in Svalbard rock ptarmigan and the difficulties of interspecies and interindividual variability, the results will inform future experimental procedures and improve scientific accuracy along with animal welfare. 

## Data Availability

The raw data used and/or analyzed in this pilot study are available from the lead author on reasonable request.

## Abbreviations

BDA: biotin dextran amine
DAB: diaminobenzidine
i.m.: intramuscular
i.v.: intravenous
PBS: phosphate–buffered saline
p.o.: peroral
s.c.: subcutaneous
SpO_2_: oxygen saturation
TBS: Tris–buffered saline
SRP: Svalbard rock ptarmigan
